# The sphingosine kinase inhibitor SKI-V suppresses cervical cancer cell growth

**DOI:** 10.7150/ijbs.71381

**Published:** 2022-04-18

**Authors:** Yan Zhang, Long Cheng, Xin Shi, Yu Song, Xiao-yu Chen, Min-bin Chen, Jin Yao, Zhi-qing Zhang, Shang Cai

**Affiliations:** 1 Department of Radiotherapy and Oncology, Affiliated Kunshan Hospital of Jiangsu University, Kunshan, China.; 2 Department of Interventional Radiology, Dushu Lake Hospital Affiliated to Soochow University, Medical Center of Soochow University, Suzhou Dushu Lake Hospital, Suzhou, China.; 3 Department of Neurology and Clinical Research Center of Neurological Disease, The Second Affiliated Hospital of Soochow University, Suzhou, China.; 4 Department of Oncology, The Affiliated Zhangjiagang Hospital of Soochow University, Suzhou, China.; 5 Changshu Hospital Affiliated to Nanjing University of Chinese Medicine, Changshu, China.; 6 The Affiliated Eye Hospital, Nanjing Medical University, Nanjing, China.; 7 Department of Radiotherapy & Oncology, The Second Affiliated Hospital of Soochow University, Institute of Radiation Oncology, Soochow University, Suzhou, China.

**Keywords:** Cervical cancer, sphingosine kinase (SphK), SKI-V, Akt-mTOR, Cancer growth.

## Abstract

Overexpression and/or overactivation of sphingosine kinase 1/2 (SphK1/2) is important for tumorigenesis and progression of cervical cancer. The current study examined the potential activity and signaling mechanisms of SKI-V, a non-lipid small molecule SphK inhibitor, against cervical cancer cells. In different primary and immortalized cervical cancer cells, SKI-V exerted significant anti-cancer activity by inhibiting cell viability, colony formation, proliferation, cell cycle progression and cell migration. Significant apoptosis activation was detected in SKI-V-treated cervical cancer cells. Significantly, SKI-V also provoked programmed necrosis cascade in cervical cancer cells, as it induced mitochondrial p53-cyclophilin-D-adenine nucleotide translocator-1 (ANT1) complexation, mitochondrial membrane potential collapse, reactive oxygen species production and the release of lactate dehydrogenase into the medium. Further, SKI-V blocked SphK activation and induced ceramide accumulation in primary cervical cancer cells, without affecting SphK1/2 expression. SKI-V-induced cytotoxicity in cervical cancer cells was largely inhibited by sphingosine-1-phosphate or the SphK1 activator K6PC-5, but was sensitized by adding the short-chain ceramide C6. Moreover, SKI-V inhibited Akt-mTOR (mammalian target of rapamycin) activation in primary cervical cancer cells, and its cytotoxicity was mitigated by a constitutively-active Akt. *In vivo*, daily intraperitoneal injection of SKI-V significantly inhibited subcutaneous primary cervical cancer xenograft growth in nude mice. Together, the SphK inhibitor SKI-V suppresses cervical cancer growth *in vitro* and *in vivo*.

## Introduction

Cervical cancer is one of the most common types of cancer and the third-most common cause of death from cancer among women 1, 2. It is responsible for about 8-10% of all cancer deaths of women every year 3, 4. Human papillomavirus (HPV) infection is associated with over 90% of all cervical cancer cases 5. The widespread use of cervical screening 6, along with other measurers, has significantly reduced the mortality, and the overall five-year survival rate is close to 72% 3, 4. The current treatments for cervical cancer include surgery, radiation therapy (used in all stages), cisplatin-based chemotherapy, and molecularly-targeted therapies 7, 8. The prognosis of metastatic, recurrent and other advanced cervical cancers remains extremely poor. Indeed, the five year survival rate decrease to 30-40% for women with stage III cervical cancers and only 15% or fewer for those with stage IV cancers 4.

Due to the limited success with current therapies for the management of advanced cervical cancer, there is increased interest in the development of novel targeted therapeutics 9, 10. Sphingosine kinases (SphKs), including SphK1 and SphK2, are conserved lipid kinases that catalyze formation sphingosine-1-phosphate (S1P) from the precursor sphingolipid 11-15. Sphingolipid metabolites, ceramide, sphingosine and S1P 11-15, are vital lipid second messengers involved in diverse cellular processes, including apoptosis regulation, cell proliferation and cell survival as well as cell migration, invasion, metastasis, and tumor neovascularization 11-15.

Kim *et al.,* have shown that SphK1 expression is dramatically increased in cervical cancer tissues and cell lines 16. Moreover, SphK1 upregulation is associated with tumor size, invasion depth, lymph node metastasis, stage, and lymphovascular invasion 16. Importantly, elevated SphK1 expression also correlated with poor prognosis and is an independent prognostic factor for predicting poor recurrence-free survival 16. Xu *et al.,* demonstrated that SphK2 expression is elevated in cervical cancer cells 17. ABC294640, a specific SphK2 inhibitor 18, 19, potently inhibited cervical cancer cell growth *in vitro* and *in vivo* 17. These evidences supported that targeting SphK1/2 could achieve significant activity against cervical cancer cells.

SKI-V (CAS No. 24418-86-8) is a non-competitive and non-lipid small molecule SphK inhibitor, and the IC_50_ is close to 2 μM for SphK 20, 21. SKI-V potently suppressed SphK activity and depleted S1P, inducing apoptosis in bladder cancer cells 21. In immunocompetent BALB/c mice, SKI-V intraperitoneal injection arrested growth of the mammary adenocarcinoma xenograft 21. The SphK inhibitor was relative safe and no significant toxicity was detected at doses up to 75 mg/kg in Swiss-Webster mice and BALB/c nude mice 21. The potential activity and the underlying mechanisms of SKI-V against cervical cancer cells were examined in the present study.

## Materials and methods

**Chemicals and reagents.** SKI-V was obtained from MedChemExpress (Beijing, China). JC-1, EdU (5-Ethynyl-2'-deoxyuridine), DAPI (4',6-diamidino-2-phenylindole), TUNEL (Terminal deoxynucleotidyl transferase dUTP nick end labeling) and CellROX dyes, as well as Annexin V and propidium iodide (PI) were purchased from Thermo-Fisher Invitrogen Co. (Shanghai, China). Antibodies were all purchased from Cell Signaling Technologies (Beverly, MA). Cell culture reagents were obtained from Hyclone (Logan, UT). Puromycin, polybrene, N-acetyl-L-cysteine (NAC), z-DEVD-fmk, z-VAD-fmk, sphingosine 1-phosphate (S1P), cyclosporin A, LY294002, PD98059 and U0126 were from Sigma-Aldrich (St. Louis, Mo). K6PC-5, SKI-II, FTY720, and ABC294640 were obtained from Selleck (Beijing, China). C6 ceramide and S1P were described in our previous studies 19, 22.

**Cell culture.** A total of three female patients (pCCa-1, pCCa-2 and pCCa-3) with gynecology and obstetrics (FIGO) stages IIA-IIB cervical cancers (squamous cell carcinoma), at age 49/55/67, were enrolled in authors institutions. The written-informed consent was provided from each patient. All patients underwent standardized treatments, including radical hysterectomy, concurrent chemo-radiation and chemotherapy. The surgery-isolated fresh tumor tissue specimens were washed with PBS and minced into small pieces. The tissues were then incubated in phenol-red free DMEM/F12 medium plus type I collagenase and DNase I for 3h. The obtained primary cells were filtrated through the nylon cell strainer (BD) 23. Cells were then centrifuged, collected, washed and resuspended in phenol-red free DMEM/F12 plus 10% FBS. Fibroblasts, blood vessel cells, immune cells and other non-cancerous cells in the medium supernatant were immediately removed after cancer cell attachment. The exact same procedure was applied to adjacent normal cervical tissues to obtain the primary human cervical epithelial cells (HCerEpC). HeLa cervical cancer cell line and Ect1/E6E7 cervical epithelial cell line were purchased from the Cell Bank of Shanghai Institute of Biological Science of CAS (Shanghai, China). The immortalized cells were cultured in high glucose (17.51 mM) DMEM/F-12 medium plus 10% FBS (Gibco, Suzhou, China). The protocols were approved by the Ethics Committee of Soochow University, in accordance to the Declaration of Helsinki.

**Quantitative real-time reverse transcriptase polymerase chain reaction (qRT-PCR) assay.** As described 24, 25, TRIzol reagents were added to cells or fresh tissues. Total RNA was reversely transcripted to cDNA. qRT-PCR assays were carried out by the ABI7600 Prism system using the SYBR Green PCR kit, and the product melting temperature was calculated. Quantification of targeted mRNA was through the 2^-∆∆Ct^ method^24^, ^26^. *GAPDH* mRNA was tested as the internal control. All verified primers were purchased from Genechem (Shanghai, China).

**Western blotting and co-immunoprecipitation.** As described 24, 25, aliquots of 30-40 μg proteins per treatment were separated by SDS-polyacrylamide gel electrophoresis (SDS-PAGE) and transferred to polyvinylidene difluoride (PVDF) membranes (Millipore, Bedford, MA). Western blotting protocols were reported previously 25, 27-29, and the ImageJ software was utilized for data quantification. The mitochondria fraction lysates were achieved using the described protocol 30, 31 and lysates (500 μg per treatment) were pre-cleared and incubated with anti-Cyclophilin-D (CyPD) antibody. The proteins that were immunoprecipitated with CyPD were captured and tested by Western blotting analyses.

**The SphK activity assay.** Following treatment, cells and tissues were homogenized and centrifuged, and the supernatant was obtained. The SphK activity was measured by the described protocol 32.

**Ceramide assay**. Cells were plated into six-well plates and treated with SKI-V. The detailed protocols of analyzing total cellular ceramides were described previously 28, 33, 34. Ceramides were expressed as fmol by nmol of phospholipid.

**Cyclophilin-D (CyPD) shRNA**. CyPD shRNA lentiviral particles were purchased from Santa Cruz Biotech and were added to cultured cervical cancer cells for 24h. Thereafter, puromycin (2.0 μg/mL) was added to select stable cells for 4-5 passages. CyPD silencing in stable cells was verified by Western blotting.

**Constitutively-active mutant Akt1.** The recombinant adenoviral construct encoding the constitutively-active Akt1 (caAkt1, S473D) was provided by Dr. Xu 35, which was stably transduced to pCCa-1 cells. Cells were then distributed into 192-well plates, and single stable cells with caAkt1 were verified by Western blotting.

**Cellular function assays**, including Cell Counting Kit-8 (CCK-8) assay of cell viability, [H^3^] DNA incorporation ELISA (enzyme-linked immunosorbent assay), nuclear EdU staining, colony formation, Trypan blue staining of cell death, propidium iodide (PI)-FACS (fluorescence-activated cell sorting) detection of cell cycle progression, “Transwell” assays were described in detail in our previous studies 27, 36, 37. Cell apoptosis-related assays, including the caspae-3 activity assay, the nuclear TUNEL staining, Annexin V-PI FACS, were described in detail in our previous studies 27, 36, 37. Cell necrosis detection by measuring medium LDH contents was described elsewhere 38.

**Mouse xenograft studies.** Animal protocols in the present study were approved by IACUC and Ethics Board of Soochow University. Five to six week-old BALB/c nude mice (half male half female, 18.3-19.5g) were purchased from the Animal Center of Soochow University and were maintained indoors at standard conditions. pCCa-1 cells (5 × 10^6^ cells per mouse, in 200 μL DMEM/Matrigel solution, no serum) were subcutaneously (*s.c.*) injected into the flanks of the nude mice. pCCa-1 xenograft tumors were then established with 20 days of cell inoculation, and each xenograft tumor was close to 100 mm^3^, and it was the time when treatment was initiated. The mice body weights and tumor volumes were measured every six days using the digital calipers 28.

**Statistical analysis.** Data were normally distributed and were presented as mean ± standard deviation (SD). Statistical analyses were carried out by SPSS 23.0 (SPSS Co., Chicago, IL). Unpaired student's T-test was employed to compare two groups. One-way ANOVA with the Scheffe' and Tukey Test was employed for comparison of multiple groups. ***P*** values of <0.05 were considered as statistically significant.

## Results

### SKI-V exerts significant anti-cancer activity in cultured cervical cancer cells

**Figure 1:**
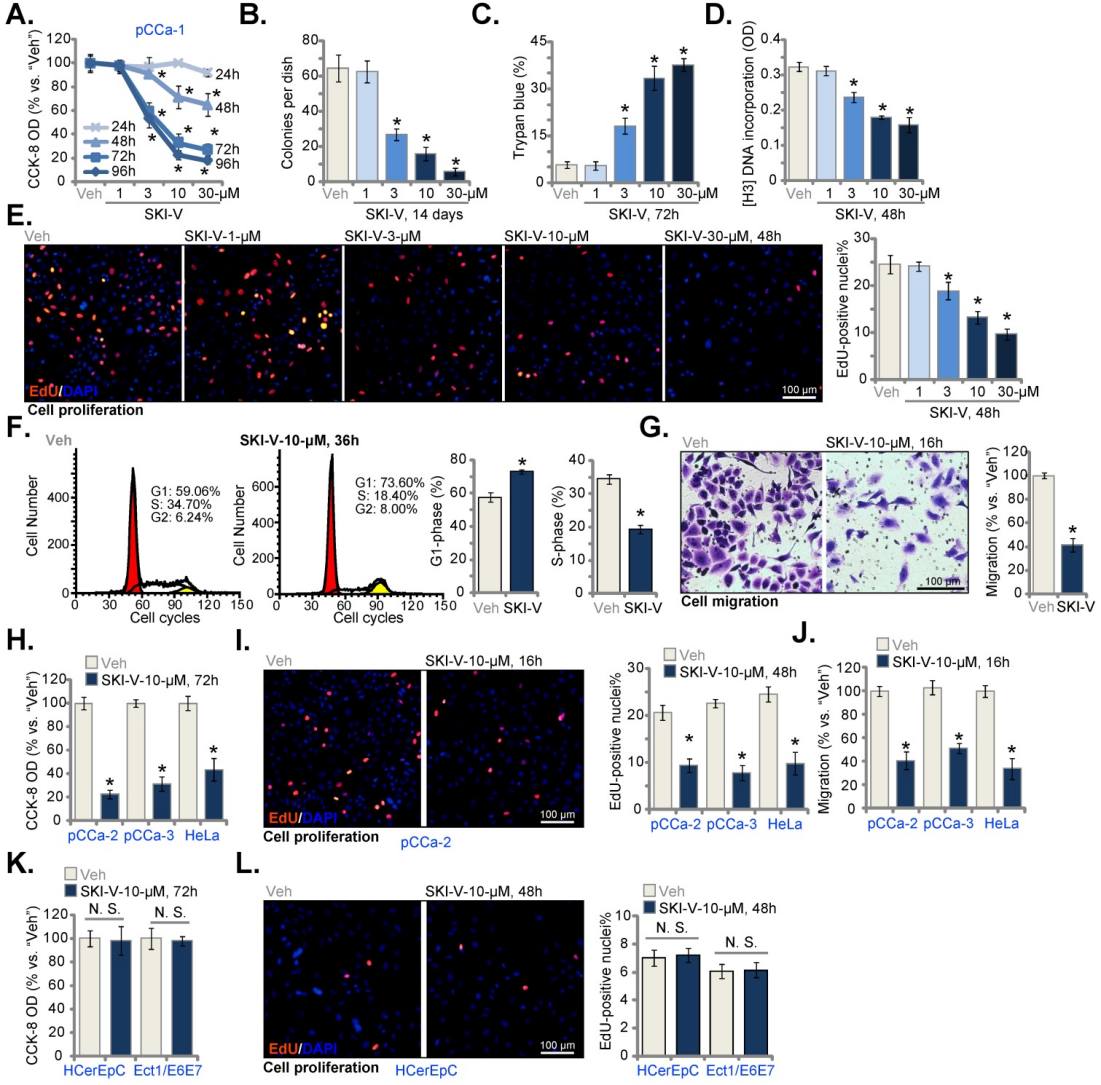
** SKI-V exerts significant anti-cancer activity in cultured cervical cancer cells.** Patient-derived primary human cervical cancer cells (pCCa-1, pCCa-2, and pCCa-3, from three patients) (**A**-**J**), the established HeLa cell line (**H**-**J**), the primary human cervical epithelial cells (HCerEpC) (**K** and **L**) or Ect1/E6E7 cervical epithelial cell line(**K** and **L**) were cultivated in FBS-containing complete medium and treated with SKI-V at the applied concentrations. Control cells were treated with the vehicle control (0.1% DMSO, “Veh”). Cells were further cultured in the conditional medium for the applied time periods, cell viability (CCK-8 OD, **A** , **H** and **K**), colony formation (**B**), cell death (by testing Trypan blue ratio, **C**) and cell proliferation (by measuring [H^3^] DNA incorporation and the EdU-positive nuclei ratio **D**, **E**, **I** and **L**) as well as cell cycle progression (PI-FACS assays, **F**) and cell migration (“Transwell” assays, **G** and **J**) were tested. For EdU staining assays, five random views of total 1, 500 cell nuclei per treatment were included to calculate the average EdU ratio (% vs. DAPI). For all “Transwell” assays, five random microscopy views of each condition were included to calculate the average number of migrated cells. Data were presented as mean ± standard deviation (SD, n=5). * ***P*** < 0.05 vs. “Veh” treatment. “N.S.” stands for the non-statistical difference (***P*** > 0.05, **K** and **L**). The experiments were repeated five times with similar results obtained. Scale bar = 100 μm (**E**, **G**, **I** and **L**).

The primary human cervical cancer pCCa-1 cells were cultivated in complete medium and treated with SKI-V at gradually-increased concentrations (from 1 to 30 μM). CCK-8 assays were carried out to examine cell viability and results demonstrated that SKI-V inhibited pCCa-1 cell viability in a concentration-dependent manner (Figure **1A**). The viability (CCK-8 OD) reduction by SKI-V was significant at 3-30 μM concentrations, but not at 1 μM (Figure **1A**). In addition, the SphK inhibitor showed a time-dependent response in decreasing pCCa-1 cell viability (Figure **1A**), as it required at least 48h to cause a significant effect (Figure **1A**) and lasted for at least 96h (Figure **1A**). The colony formation assay results in Figure **1B** further supported the anti-survival activity of SKI-V in pCCa-1 cells, and the significantly decreased number of pCCa-1 cell colonies was observed following SKI-V (3-30 μM) treatment (Figure **1B**). The positive trypan blue staining is a characteristic marker of cell death. SKI-V, in a concentration-dependent response, increased the number of trypan blue-positive pCCa-1 cells, supporting its cytotoxic effect to cervical cancer cells (Figure **1C**).

Further experimental results showed that SKI-V (3-30 μM) treatment significantly decreased [H^3^] DNA incorporation (ELISA OD) in pCCa-1 cells (Figure **1D**), suggesting that the SphK inhibitor suppressed cervical cancer cell proliferation. Moreover, the EdU-positive nuclei ratio in pCCa-1 cells was robustly decreased following SKI-V (3-30 μM) treatment (Figure **1E**), further supporting the anti-proliferative activity of SKI-V. The titration experiments in Figure **1A**-**E** showed that SKI-V at 10 µM resulted in significant anti-cervical cancer activity and this concentration was selected for the following experiments.

The PI-FACS assays were then carried out to study the potential effect of SKI-V on cell cycle progression. Results implied that SKI-V (10 μM) resulted in G1-S cell cycle arrest in pCCa-1 cells (Figure **1F**), causing increased G1-phase cell ratio but decreased S-phase cell ratio (Figure **1F**). The *in vitro* cell mobility assay results showed that SKI-V (10 μM) potently inhibited pCCa-1 cell migration, tested by the “Transwell” assay (Figure **1G**).

Whether SKI-V could exert significant anti-cancer activity in other cervical cancer cells was studied. The primary cervical cancer cells that were derived from two other patients, pCCa-2 and pCCa-3, as well as the immortalized HeLa cells were cultivated and treated with SKI-V (10 μM). The SphK inhibitor resulted in significant cytotoxicity in the cervical cancer cells, leading to robust viability (CCK-8 OD) reduction (Figure **1H**). Moreover, in the cervical cancer cells, SKI-V largely inhibited cell proliferation (tested by EdU-positive nuclei ratio, Figure **1I**) and migration (Figure **1J**). In the primary human cervical epithelial cells (HCerEpC) and immortalized Ect1/E6E7 epithelial cells, the very same SKI-V treatment failed to significantly inhibit cell viability (CCK-8 OD, Figure **1K**) and proliferation (tested by EdU staining assays, Figure **1L**), indicating a cancer cell specific effect of the SphK inhibitor.

### SKI-V provokes apoptosis in cervical cancer cells

**Figure 2:**
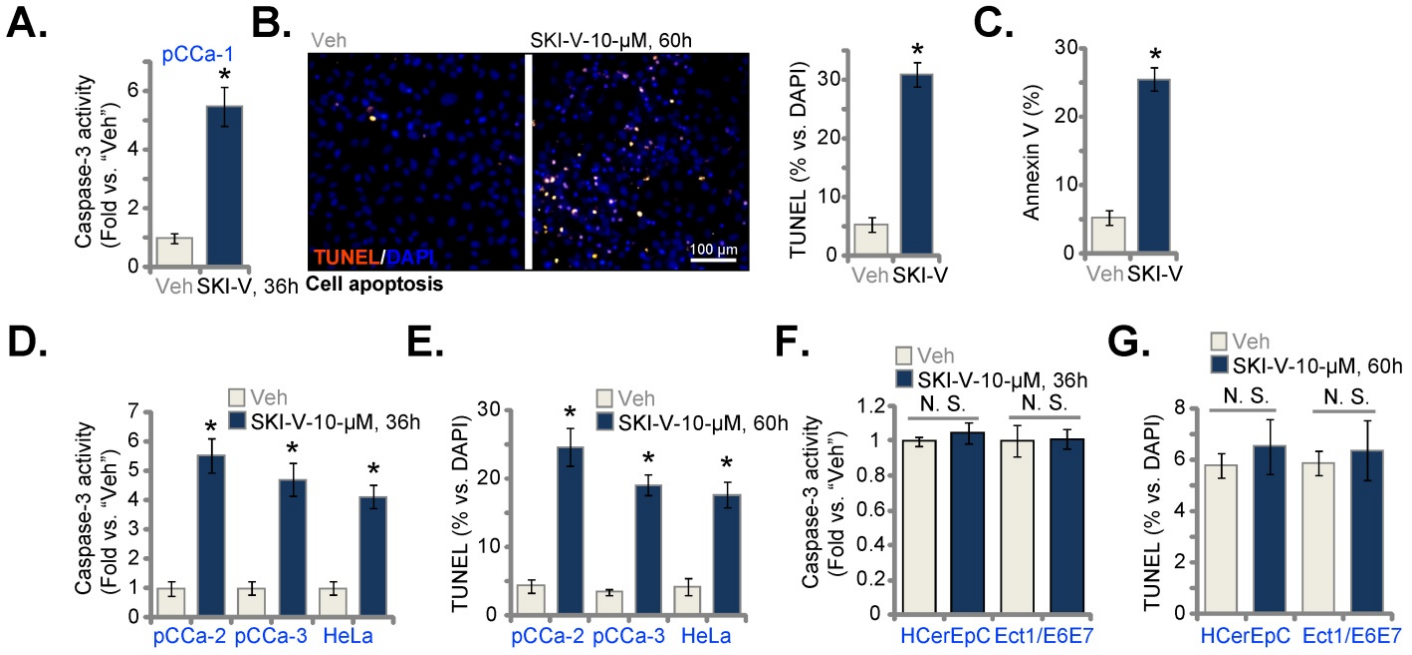
** SKI-V provokes apoptosis in cervical cancer cells.** Patient-derived primary human cervical cancer cells (pCCa-1, pCCa-2, and pCCa-3) (**A**-**E**), the established HeLa cell line (**D** and **E**), the primary human cervical epithelial cells (HCerEpC) (**F** and **G**) or Ect1/E6E7 cervical epithelial cell line (**F** and **G**) were cultivated in FBS-containing complete medium and treated with SKI-V (10 μM). Control cells were treated with the vehicle control (0.1% DMSO, “Veh”). Cells were further cultured in the conditional medium for the applied time periods, caspase-3 activation (**A**, **D** and **F**) was tested. Cell apoptosis was tested by nuclear TUNEL staining (**B**, **E** and **G**, with results quantified) and Annexin V FACS (**C**) assays. Data were presented as mean ± standard deviation (SD, n=5). * ***P*** < 0.05 vs. “Veh” treatment. “N.S.” stands for the non-statistical difference (***P*** > 0.05, **F** and **G**). Scale bar = 100 μm (**B**).

SphK inhibition will cause S1P depletion and ceramide accumulation, leading to cell apoptosis 11-14. We therefore tested whether SKI-V could provoke apoptosis in pCCa-1 cervical cancer cells. As shown in Figure **2A**, following SKI-V (10 μM) treatment, the caspase-3 activity was dramatically enhanced. Significant apoptosis was detected and SKI-V robustly increased the TUNEL-positive nuclei ratio (Figure **2B**) and Annexin V-positive staining (Figure **2C**) in pCCa-1 cells. Significantly, SKI-V provoked apoptosis activation in other cervical cancer cells as well. The SphK1 inhibitor significantly increased the caspase-3 activity (Figure **2D**) and the TUNEL-positive nuclei ratio (Figure **2E**) in primary pCCa-2 and pCCa-3 cells as well as in the established HeLa cells. These results demonstrated that SKI-V induced apoptosis in cervical cancer cells. In HCerEpC and Ect1/E6E7 epithelial cells, treatment with SKI-V however failed to significantly increase the caspase-3 activity (Figure **2F**) and TUNEL-positive nuclei ratio (Figure **2G**), again showing the cancer cell specific effect.

### SKI-V provokes programmed necrosis in cervical cancer cells

**Figure 3:**
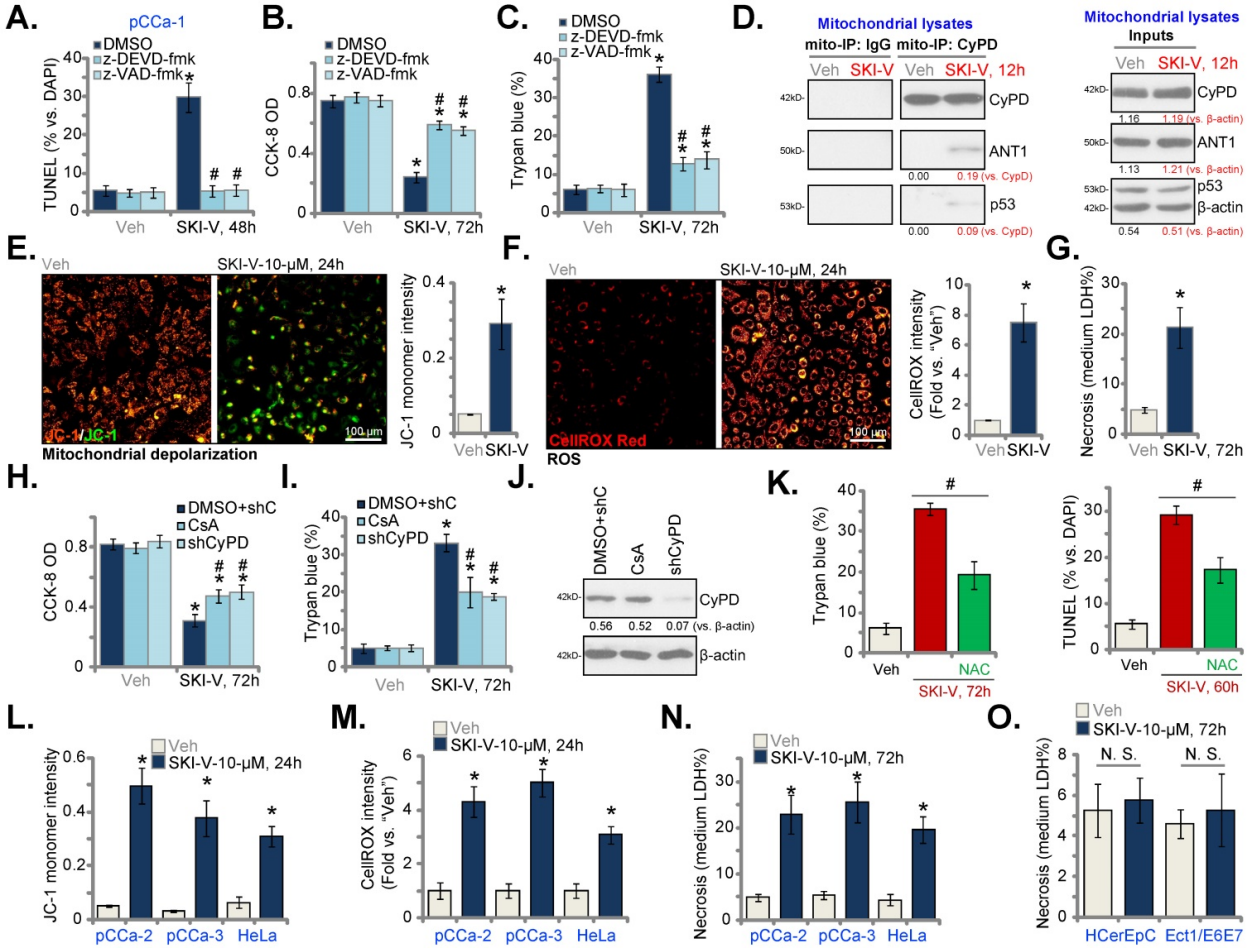
**SKI-V provokes programmed necrosis in cervical cancer cells.** The pCCa-1 primary human cervical cancer cells were pretreated for 1h with the caspase-3 inhibitor z-DEVD-fmk (40 μM), the pan caspase inhibitor z-VAD-fmk (40 μM) or the vehicle control, followed by SKI-V (10 μM) treatment; Cells were further cultured in the conditional medium for the applied time periods, cell apoptosis, viability and death were tested by nuclear TUNEL staining (**A**), CCK-8 (**B**) and Trypan blue staining (**C**) assays, respectively. Patient-derived primary human cervical cancer cells (pCCa-1, pCCa-2, and pCCa-3) (**D**-**G**, **L**-**N**), the established HeLa cell line (**L**-**N**), the primary human cervical epithelial cells (HCerEpC) (**O**) or Ect1/E6E7 cervical epithelial cell line (**O**) were treated with SKI-V (10 μM) or the vehicle control (0.1% DMSO, “Veh”) for applied time periods, mitochondrial p53-CyPD-ANT1 association was tested by mitochondrial immunoprecipitation (mito-IP) assays (**D**), their expression was examined as well (**D**, “Inputs”); Mitochondrial membrane potential (MMP) reduction, ROS production and cell necrosis were tested by JC-1 staining (**E** and **L**), CellROX staining (**F** and **M**) and medium LDH release (**G**, **N** and **O**) assays, respectively. The pCCa-1 cells expressing the CyPD shRNA (“shCyPD”), with cyclosporin A (“CsA”, 10 μM) pretreatment (for 1h) or with scramble control shRNA plus 0.1% DMSO treatment (“DMSO+shC”) were treated with SKI-V (10 μM) or the vehicle control; Cells were further cultured for the applied time periods, cell viability and death were tested by CCK-8 (**H**) and Trypan blue staining (**I**) assays, respectively. Expression of listed proteins was shown (**J**). The pCCa-1 primary cells were pretreated with N-acetyl-L-cysteine (NAC, 500 μM) for 30 min, followed by SKI-V (10 μM) stimulation, and cells were cultured for applied time periods, and cell death (by measuring Trypan blue-positive cell ratio) and apoptosis (by measuring TUNEL-positive nuclei ratio) were tested (**K**). Data were presented as mean ± standard deviation (SD, n=5). * ***P*** < 0.05 vs. “Veh” treatment. ^#^ ***P*** < 0.05 vs. “DMSO” pretreatment (**A**-**C**). ^#^ ***P*** < 0.05 (**K**). ^#^ ***P*** < 0.05 vs. “DMSO+shC” cells (**H** and **I**). “N.S.” stands for the non-statistical difference (***P*** > 0.05, **O**). Scale bar = 100 μm (**E** and **F**).

To examine whether apoptosis was the only mechanism of SKI-V-induced cytotoxicity in cervical cancer cells, the apoptosis inhibitors were applied, including the caspase-3 inhibitor z-DEVD-fmk and the pan caspase inhibitor z-VAD-fmk. The nuclear TUNEL staining assay results demonstrated that the two caspase inhibitors blocked apoptosis activation (evidenced by the TUNEL staining assays) in the pCCa-1 primary cervical cancer cells (Figure **3A**). However, the caspase inhibitors only partially attenuated SKI-V-induced viability (CCK-8 OD) reduction (Figure **3B**) and cell death (Figure **3C**). These results implied that besides apoptosis, the SphK inhibitor could also induce other forms of cell death in cervical cancer cells.

Recent studies have identified a mitochondria-dependent pathway of programmed necrosis, 39-43. A number of anti-cancer agents could provoke the programmed necrosis cascade, contributing to cancer cell death 44-48. The mitochondrial immunoprecipitation (mito-IP) assay results in Figure **3D** showed that SKI-V treatment induced p53-CyPD-adenine nucleotide translocator-1 (ANT1) complexation in the mitochondria of pCCa-1 cells, known as the initial step of programmed necrosis cascade induction 39, 49, 50. The SphK inhibitor also induced mitochondrial membrane potential (MMP) collapse in pCCa-1 cells, evidenced by JC-1 green monomer accumulation (Figure **3E**). Reactive oxygen species (ROS) levels were tested by CellROX intensity assays, and results found that ROS were significantly increased in SKI-V-treated pCCa-1 cells (Figure **3F**). The increased lactate dehydrogenase (LDH) releasing in the medium confirmed necrosis-induced by SKI-V in pCCa-1 cells (Figure **3G**). Importantly, cyclosporin A (CsA), the CyPD inhibitor 48, 51, or shRNA-induced silencing of CyPD (“shCyPD”) inhibited SKI-V-induced viability (CCK-8 OD) reduction (Figure **3H**) and cell death (Figure **3I**). CyPD silencing was verified by Western blotting assays (Figure **3J**). The antioxidant N-acetyl-L-cysteine (NAC) attenuated SKI-V-induced pCCa-1 cell death and apoptosis in pCCa-1 primary cells (Figure **3K**).

In pCCa-2 and pCCa-3 primary cells as well as in the established HeLa cells, treatment with SKI-V similarly induced MMP reduction (tested by JC-1 green monomer accumulation, Figure **3L**), ROS production (tested by CellROX intensity increase, Figure **3M**) and cell necrosis (LDH releasing to the medium, Figure **3N**). In HCerEpC and immortalized Ect1/E6E7 epithelial cells, the very same SKI-V treatment however failed to induce cell necrosis and medium LDH contents were not significantly changed (Figure **3O**). These results indicated that programmed necrosis cascade activation contributed to SKI-V-induced cytotoxicity in cervical cancer cells.

### SKI-V inhibits SphK in cervical cancer cells

**Figure 4:**
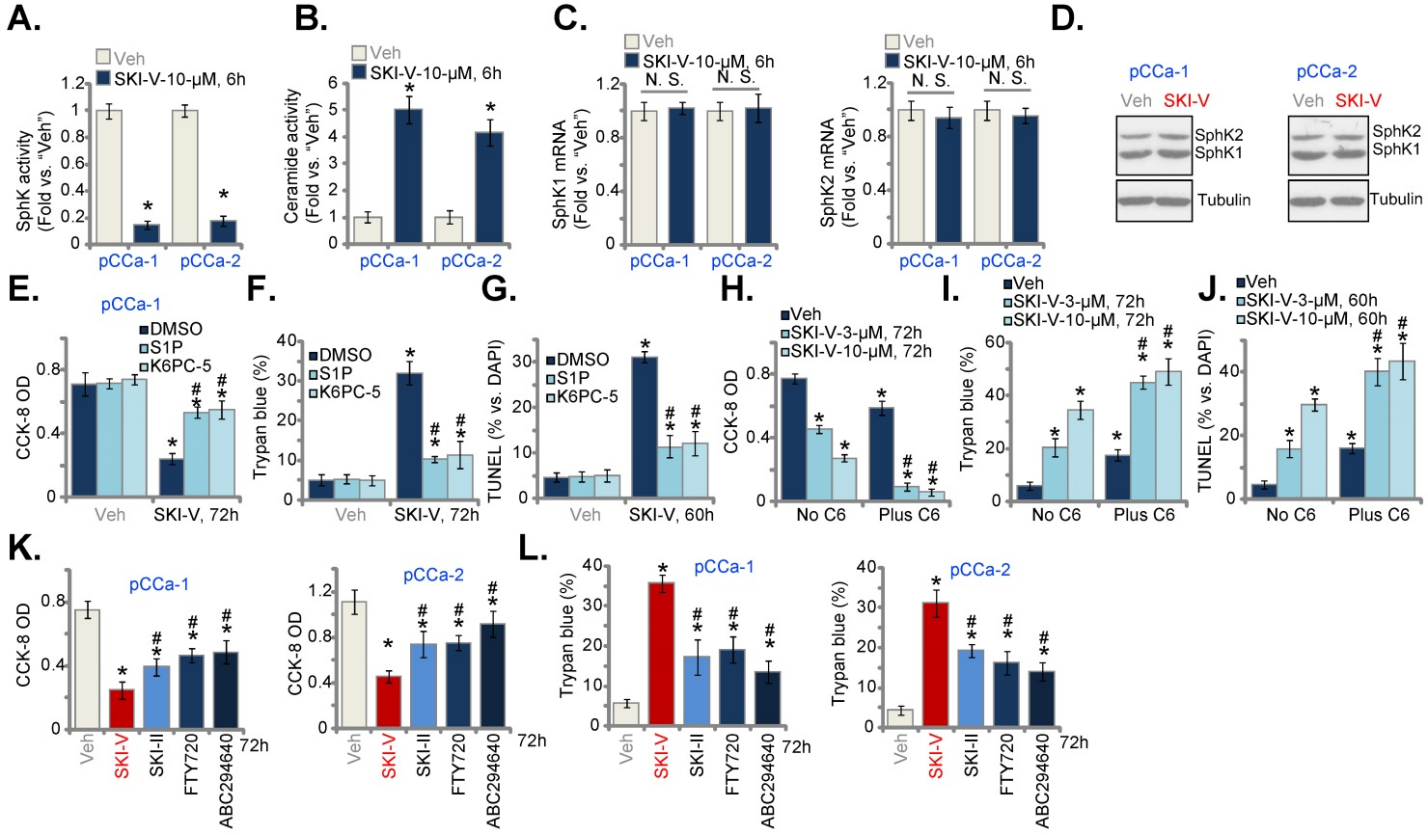
** SKI-V inhibits SphK in cervical cancer cells.** Patient-derived primary human cervical cancer cells (pCCa-1 and pCCa-2) were treated with SKI-V (10 μM) or the vehicle control (0.1% DMSO, “Veh”). Cells were further cultured in the conditional medium for the applied time periods, the SphK activity (**A**), cellular ceramide contents (**B**) and expression of SphK1/2 (both mRNA and protein, **C** and **D**) were shown. pCCa-1 cervical cancer cells were pretreated for 1h with S1P (10 μM), K6PC-5 (10 μM) or 0.1% DMSO, followed by SKI-V (10 μM) treatment and cultured for applied time periods, cell viability, death and apoptosis were tested by CCK-8 (**E**), Trypan blue staining (**F**) and nuclear TUNEL staining (**G**),assays, respectively. pCCa-1 cells were treated with SKI-V (3/10 μM), or together with C6 ceramide (10 μg/mL), cells were further cultured for the applied time periods, and cell viability (**H**), death (**I**) and apoptosis (**J**) were tested similarly. pCCa-1 or pCCa-2 cells were treated with 10 μM of SKI-V, SKI-II, FTY720 or ABC294640 for the applied time periods, cell viability and death were tested by CCK-8 (**K**) and Trypan blue staining (**L**) assays, respectively. Data were presented as mean ± standard deviation (SD, n=5). * ***P*** < 0.05 vs. “Veh” treatment. ^#^ ***P*** < 0.05 vs. “DMSO” pretreatment group (**E**-**G**). ^#^ ***P*** < 0.05 vs. no C6 ceramide co-treatment (**H**-**J**). ^#^ ***P*** < 0.05 vs. SKI-V treatment (**K** and **L**). “N.S.” stands for the non-statistical difference (***P*** > 0.05, **C**).

Next, we tested whether SKI-V indeed blocked SphK activation. In pCCa-1 and pCCa-2 primary cervical cancer cells, treatment with SKI-V (10 μM, 6h) robustly decreased the SphK activity (Figure 4A), and induced ceramide accumulation (Figure 4B). The mRNA (Figure 4C) and protein (Figure 4D) expression of SphK1 and SphK2 were however unchanged after SKI-V treatment. Importantly, exogenously adding S1P or treatment with the SphK1 activator K6PC-5 52-54 largely inhibited SKI-V-induced viability (CCK-8 OD) reduction (Figure 4E), cell death (Trypan blue assays, Figure 4F) and apoptosis (tested by nuclear TUNEL staining, Figure 4G) in pCCa-1 cells. On the contrast, a short-chain ceramide C6 22, 55, 56 augmented SKI-V-induced cytotoxicity and apoptosis in pCCa-1 cells (Figure 4H-J). These results implied that SKI-V-induced cytotoxicity in cervical cancer cells was associated with SphK blockage. We also compared the anti-cervical cancer cell activity of SKI-V with other known SphK inhibitors, including SKI-II 57-59, FTY720 60, 61 and the SphK2 specific inhibitor ABC294640 18, 19, 62. As shown in pCCa-1 and pCCa-2 primary cells, SKI-V-induced cytotoxicity (viability reduction, Figure 4K) and death (tested by LDH releasing to the medium, Figure 4L) were more significant than other SphK inhibitors (at the same concentration).

### SKI-V inhibits Akt-mTOR activation in cervical cancer cells

**Figure 5:**
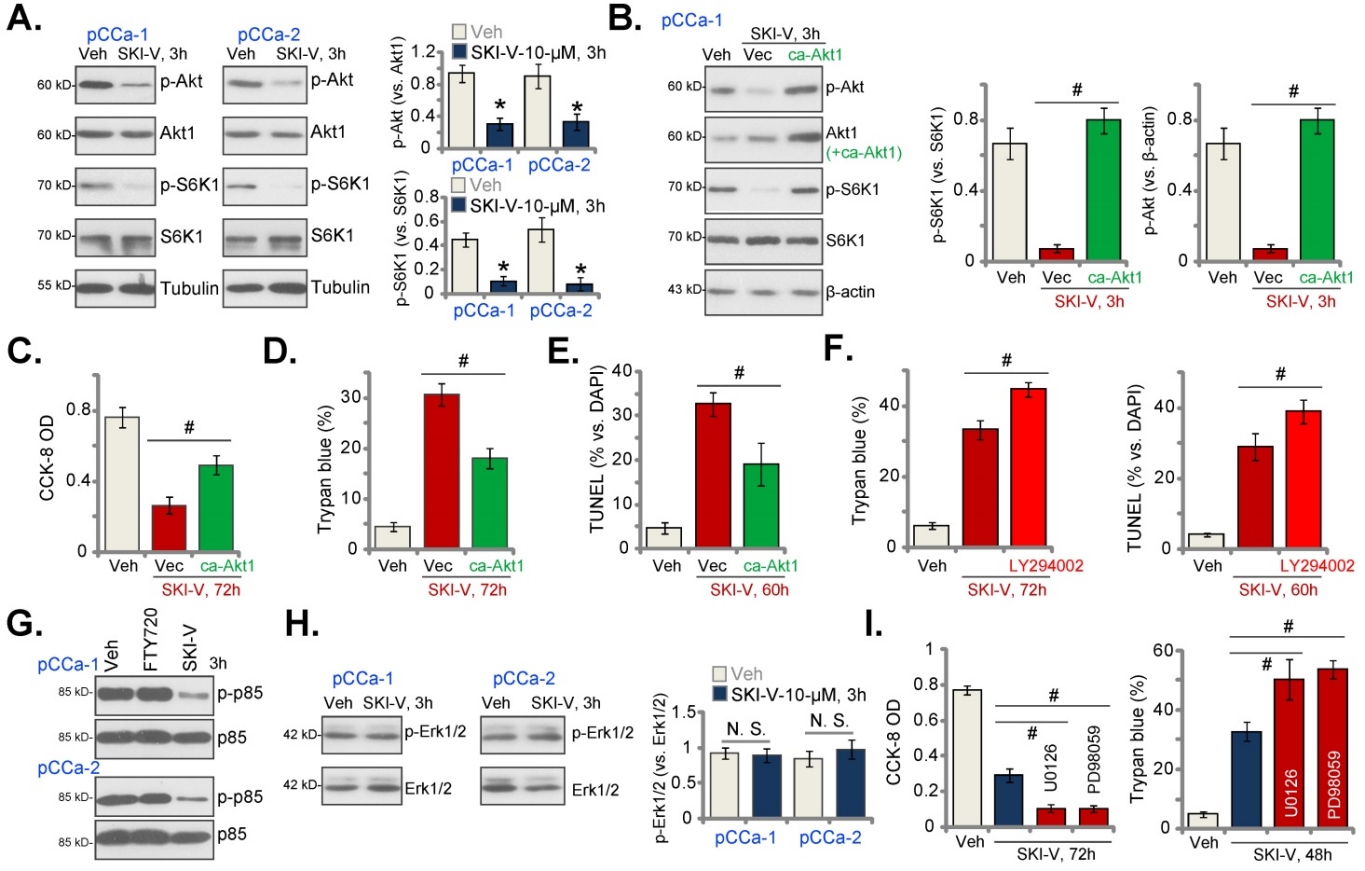
** SKI-V inhibits Akt-mTOR activation in cervical cancer cells.** Patient-derived primary human cervical cancer cells (pCCa-1 and pCCa-2) were treated with SKI-V (10 μM) or the vehicle control (0.1% DMSO, “Veh”), and cells were cultured in the conditional medium for 3h, expression of listed proteins was shown (**A** and **H**). pCCa-1 cells expressing the adenovirus-encoded constrictively-active Akt1 (“caAkt1”, S473D) construct or the empty vector (“Vec”) were treated with SKI-V (10 μM), the parental control cells were treated with the vehicle control (0.1% DMSO, “Veh”). Cells were further cultured in the conditional medium for the applied time periods, expression of listed proteins was shown (**B**); Cell viability, death and apoptosis were tested by CCK-8 (**C**), Trypan blue staining (**D**) and nuclear TUNEL staining (**E**) assays, respectively. The pCCa-1 primary cells were pretreated with LY294002 (1 μM) for 30 min, followed by SKI-V (10 μM) stimulation, and cells were cultured for applied time periods; Cell death (by measuring Trypan blue-positive cell ratio) and apoptosis (by measuring TUNEL-positive nuclei ratio) were tested (**F**). pCCa-1 cells were treated with 10 μM of SKI-V or FTY720 for 3h, p-p85 and total p85 expression was shown (**G**).pCCa-1 primary cells were pretreated with PD98059 (10 μM) and U0126 (10 μM) for 1h, followed by SKI-V (10 μM) stimulation, and cells were cultured for applied time periods; Cell viability (by measuring CCK-8 OD) and death (by measuring Trypan blue-positive cell ratio) were tested (**I**). Data were presented as mean ± standard deviation (SD, n=5). * ***P*** < 0.05 vs. “Veh” treatment (**A**). ^#^ ***P*** < 0.05 (**B**-**F** and **I**). “N.S.” stands for the non-statistical difference (***P*** > 0.05, **H**).

The results above suggested that there could be SphK inhibition-independent mechanism participating in SKI-V-induced anti-cervical cancer cell activity. Akt-mammalian target of rapamycin (mTOR) overactivation is essential for cervical cancer progression 63-65. In pCCa-1 and pCCa-2 cervical cancer cells, SKI-V (10 μM, 3h) significantly suppressed phosphorylation of Akt (Ser-473) and S6K (Thr-389) (Figure 5A), indicating that SKI-V inhibited Akt-mTOR cascade activation in cervical cancer cells. To study the association between SKI-V-induced Akt-mTOR inhibition and cytotoxicity, an adenovirus-encoded constrictively-active Akt1 (caAkt1, S473D) construct 35 was stably transduced to pCCa-1 cells, which restored Akt-S6K1 phosphorylation in SKI-V (10 μM, 3h)-treated cells (Figure 5B). Significantly, in pCCa-1 cells SKI-V-induced viability (CCK-8 OD) reduction (Figure 5C) and cell death (tested by Trypan blue staining increase, Figure 5D) were mitigated by caAkt1. Moreover, apoptosis induction in SKI-V-treated pCCa-1 cells, evidenced by increased TUNEL-positive nuclei ratio, was ameliorated by caAkt1 as well (Figure 5E).

The PI3K-Akt-mTOR pan inhibitor, LY294002 66, intensified SKI-V-induced cytotoxicity and apoptosis in pCCa-1 primary cervical cancer cells (Figure **5F**). These results implied that Akt-mTOR inactivation participated in SKI-V-induced cytotoxicity in cervical cancer cells. SKI-V potently inhibited PI3K activation (p85 phosphorylation) in pCCa-1 and pCCa-2 cervical cancer cells (Figure **5G**). Yet, FTY720 failed to inhibit p85 phosphorylation (revised Figure **5G**). Therefore, PI3K-Akt-mTOR inactivation could be the unique action by SKI-V, independent of SphK inhibition.

Interestingly, treatment with the SphK inhibitor failed to inhibit Erk-MAPK activation (Erk1/2 phosphorylation, Figure **5H**). Yet, the Erk-MAPK inhibitors, including PD98059 and U0126, augmented SKI-V-induced viability reduction (Figure **5I**) and cell death (Figure **5H**). These results implied that Erk-MAPK activation serves as an endogenous resistant mechanism for SKI-V-induced activity against cervical cancer cells, and Erk-MAPK inhibition could sensitize SKI-V-induced activity.

### SKI-V administration inhibits cervical cancer cell growth in nude mice

**Figure 6:**
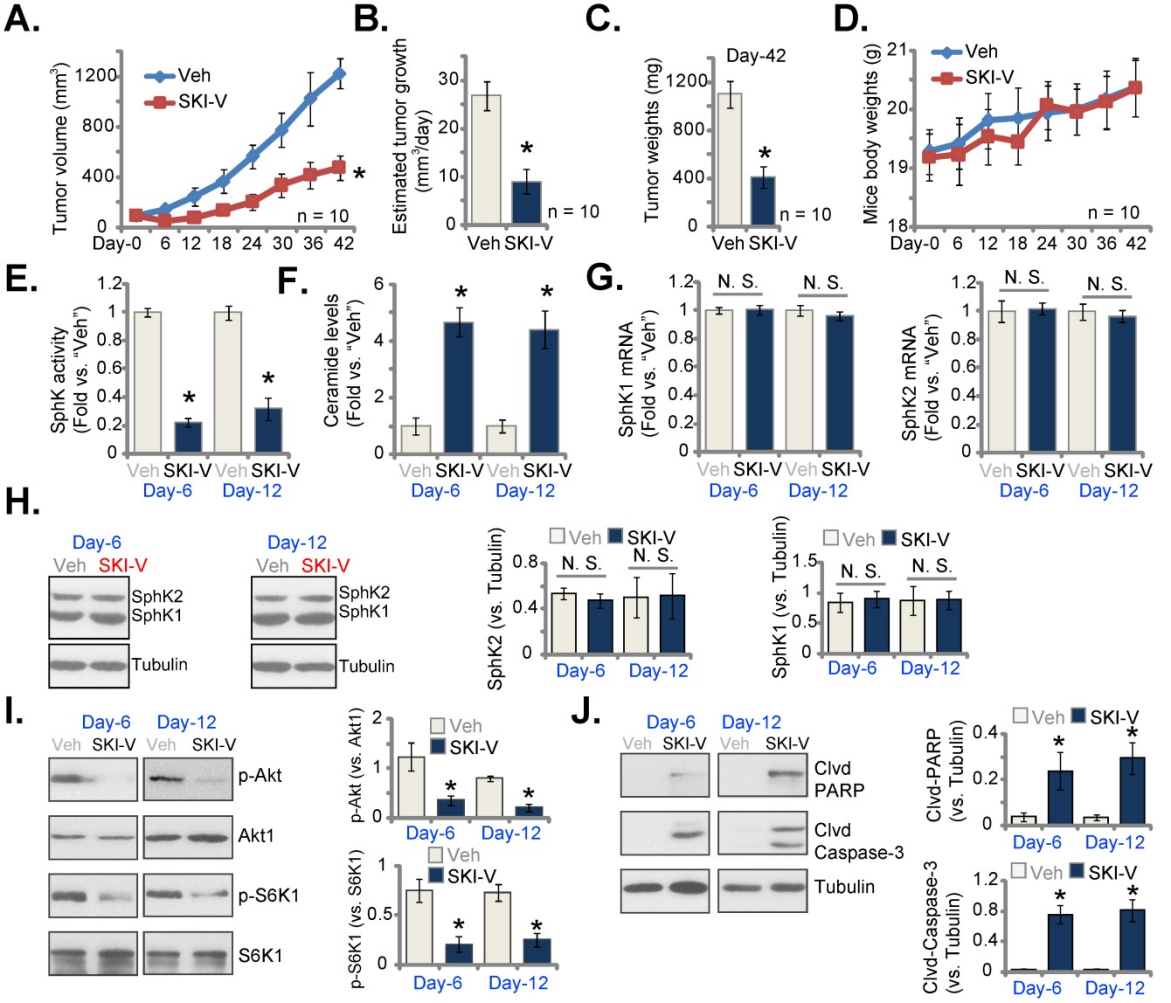
** SKI-V administration inhibits cervical cancer cell growth in nude mice.** pCCa-1 xenograft-bearing nude mice were subject to intraperitoneal (*i.p.*) injection of SKI-V (at 25 mg/kg body weight, daily for 15 days) or the vehicle control (“Veh”), the tumor volumes (**A**) and the mice body weights (**D**) were recorded every six days (“Day-0” to “Day-42”). The estimated daily tumor growth was calculated using the formula described (**B**). At “Day-42” all tumors were separated carefully and tumor weights recorded (**C**). In the described tumor tissue lysates, SphK activity (**E**), ceramide contents (**F**), expression of listed mRNAs (**G**) and proteins (**H**-**J**) were tested, with results quantified. Data were presented as mean ± standard deviation (SD). n=10 stands for 10 mice per group (**A**-**D**). For **E**-**J** expression of listed genes and proteins in five small pieces of each tumor xenograft was tested (n=5), and results were combined and quantified. * ***P*** < 0.05 vs. “Veh” treatment. “N.S.” stands for the non-statistical difference (***P*** > 0.05, **H**).

At last, we tested the potential activity of SKI-V against cervical cancer cells *in vivo*. A significant number of pCCa-1 cells (five million cells per mouse) were inoculated and subcutaneously (*s.c.*) injected to the flanks of nude mice. pCCa-1 xenograft tumors were then established with 20 days of cell inoculation, and each xenograft tumor was close to 100 mm^3^ in volume (labeled as “Day-0”). The xenograft-bearing mice were then randomly assigned into two groups, receiving intraperitoneal (*i.p.*) injection of SKI-V (at 25 mg/kg body weight, daily for 15 days) or the vehicle control (“Veh”). Figure **6A** demonstrated that SKI-V injection significantly inhibited subcutaneous pCCa-1 xenograft tumor growth in nude mice. We also calculated the estimated daily tumor growth using the described formula 67. The growth of SKI-V-treated tumors was significantly slower than vehicle administration (Figure **6B**). Xenograft tumors of the two groups were isolated at “Day-42” and weighted individually. As shown, SKI-V-treated tumors were significantly lighter than vehicle controls (Figure **6C**). There was no significant difference in the mice body weights (Figure **6D**).

At “Day-6” and “Day-12”, six hours after SKI-V/Veh administration, we carefully isolated one tumor of each group and total four xenograft tumors were obtained. The tumors were cut into small pieces and tumor tissue lysates were obtained. As shown the SphK activity in SKI-V-treated tumor tissues was dramatically decreased (Figure **6E**), while ceramide contents were significantly increased (Figure **6F**). The mRNA (Figure **6G**) and protein (Figure **6H**) expression of SphK1 and SphK2 in pCCa-1 tumor tissues were unchanged after SKI-V treatment. Importantly, Akt-mTOR activation was significantly inhibited in SKI-V-treated pCCa-1 tumor tissues, as the levels of phosphorylated Akt1 and S6K were dramatically decreased (Figure **6I**). In addition, increased cleavages of PARP and caspase-3 were detected in pCCa-1 tumor tissues with SKI-V administration, indicating apoptosis activation (Figure **6J**).

## Discussion

Cervical cancer is the third most common malignancy among women, causing over 275, 000 deaths globally each year 1, 2. The effective molecularly-targeted therapies for cervical cancer are urgently needed, particularly in developing countries 9, 10. SphKs, including SphK1 and SphK2, are important therapeutic targets of cervical cancer 16, 17. SphK inhibitors, SKI-II 57-59 and FTY720 60, 61, potently inhibited survival and induced apoptosis in established cervical cancer cells 16. Moreover, FTY720 robustly inhibited in vivo tumor growth in a patient-derived xenograft (PDX) model of cervical cancer 16.

SKI-V is a non-lipid small molecule SphK inhibitor. We found that SKI-V treatment in cultured cervical cancer cells potently inhibited SphK activity and induced ceramide accumulation. In different primary cervical cancer cells (pCCa-1/-2/-3) and established HeLa cell line, SKI-V exerted significant anti-cancer activity, as it inhibited cell viability, colony formation, proliferation, cell cycle progression (causing G1-S arrest) and cell migration. Significant apoptosis activation was detected as well in the SKI-V-treated cells. *In vivo*, daily intraperitoneal injection of a single dose of SKI-V (25 mg/kg body weight) robustly suppressed subcutaneous pCCa-1 xenograft growth in nude mice. SphK inhibition, ceramide accumulation and apoptosis induction were detected in SKI-V-treated xenograft tissues. Importantly, treatment with the SphK inhibitor failed to induce significant cytotoxicity and apoptosis in non-cancerous cervical epithelial cells. Moreover, the nude mice were well-tolerated to the SKI-V treatment regimen, showing no apparent toxicities. Therefore, SKI-V could be a promising therapeutic option with important translational value for cervical cancer.

Existing studies have shown that a number of different anti-cancer agents can induce programmed necrosis cascade in cancer cells. Zhang *et al.,* have shown that berberine induced both apoptosis and programmed necrosis in prostate cancer cells, and necrosis contributed more than apoptosis in contributing berberine-induced cytotoxicity in prostate cancer cells 48. Conversely, inhibition of CyPD-p53 cascade potently attenuated berberine-induced cytotoxicity in prostate cancer cells 48. Qin *et al.* have shown that salinomycin induced programmed necrosis cascade activation in glioma cells 47. Conversely, CyPD silencing or inhibition significantly attenuated salinomycin-induced glioma cell necrosis and cytotoxicity 47. Guo *et al.,* showed that AICAR (5-Aminoimidazole-4-carboxamide riboside or acadesine) induced ROS production and AMP-activated protein kinase (AMPK)-independent programmed necrosis cascade in prostate cancer cells 46.

In colorectal cancer (CRC) cells the SphK inhibitor PF-543 provoked programmed necrosis cascade by inducing mitochondrial p53-CyPD association, mitochondrial membrane potential reduction and the release of LDH to the medium. Conversely, CyPD silencing or inhibition largely attenuated CRC cell necrotic death by PF-543. The Cyp-D inhibitor cyclosporin A largely inhibited the *in vivo* anti-tumor activity by PF-543 in xenograft mice 45. In the present study we showed that SKI-V induced programmed necrosis cascade in cervical cancer cells as well. The SphK inhibitor induced mitochondrial p53-CyPD-ANT1 complexation, mitochondrial membrane potential collapse, ROS production and the release of LDH into the medium. CyPD inhibition (by CsA) or silencing (by targeted shRNA) mitigated SKI-V-induced cytotoxicity in primary cervical cancer cells. Therefore, besides apoptosis, simultaneous activation of the programmed necrosis cascade should be one of important reasons for the superior anti-cervical cancer cell activity by SKI-V. Indeed, we found that SKI-V was significantly more potent than other known SphK inhibitors (SKI-II, FTY720 and ABC294640) in killing cervical cancer cells.

PI3K-Akt-mTOR is often dysregulated and overactivated in cervical cancer due to various genetic mutations, including *PTEN* depletion, *PI3KCA* mutation and sustained activation or mutation of multiple receptor tyrosine kinases (RTKs) 63-65. The PI3K-Akt-mTOR cascade could be a valuable and promising therapeutic target and the biomarker predicting the prognosis of cervical cancer 63-65. Moreover, PI3K-Akt-mTOR cascade is essential for the virus/host cell crosstalk in HPV-positive cervical cancer 64. A number of different agents or genetic methods targeting this cascade can efficiently inhibit cervical cancer cell growth and induce cell death 63, 68-71. Here we found that SKI-V potently inhibited Akt-mTOR activation in primary cervical cancer cells, and restoring Akt activation by caAkt1 ameliorated SKI-V-induced cytotoxicity in cervical cancer cells. Akt-mTOR inactivation was also detected SKI-V-treated xenograft tissues. Thus, Akt-mTOR inhibition is another important mechanism of SKI-V-induced anti-cervical cancer activity.

## Conclusion

The SphK inhibitor SKI-V suppresses cervical cancer growth *in vitro* and *in vivo*. It could have important translational value for cervical cancer.
